# Know your epidemic, know your response: understanding and responding to the heterogeneity of the COVID‐19 epidemics across Southeast Asia

**DOI:** 10.1002/jia2.25557

**Published:** 2020-07-05

**Authors:** Annette H Sohn, Nittaya Phanuphak, Stefan Baral, Adeeba Kamarulzaman

**Affiliations:** ^1^ TREAT Asia/amfAR The Foundation for AIDS Research Bangkok Thailand; ^2^ Institute of HIV Research and Innovation Bangkok Thailand; ^3^ Department of Epidemiology Johns Hopkins School of Public Health Baltimore MD USA; ^4^ Faculty of Medicine Universiti Malaya Kuala Lumpur Malaysia

**Keywords:** COVID‐19, Southeast Asia, HIV, pandemic, lockdown, social distancing

As living in the midst of the COVID‐19 pandemic becomes the new normal, the heterogeneity in the burden and secondary mortality across global epidemics has become increasingly evident. This is especially notable in Southeast Asia, a region with substantial variation in population density, income levels, access to healthcare, and public health infrastructure (Table 1). It has extensive travel exchanges with East Asia, but to date has experienced relatively limited local epidemics. By mid‐May, local confirmed COVID‐19 cases varied between 19 in Lao PDR to approximately 29,000 in Singapore [1]. In several settings, efforts are now under way to lift lockdown restrictions.

**Table 1:** National demographic and reported COVID‐19 data for member countries of the Association of South East Asian Nations as of 21 May 2020

Country	Population	Density people/km^2^	GDP per capita, PPP	UHC service coverage index [14]^a^	Total reported COVID‐19 cases	COVID‐19 cases per 1 million people	Total reported COVID‐19 deaths	COVID‐19 deaths per 1 million people	Beginning of local lockdowns	Border closures/restrictions on entry of foreigners
Brunei	437,479	83	61,860	≥80	141	323	1	2	11 March	24 March
Cambodia	16,718,965	95	4262	55	122	7	0	–	28 March	30 March
Indonesia	273,523,615	151	11,647	49	19,189	70	1242	5	15 March	25 April
Lao PDR	7,275,560	32	7778	48	19	3	0	–	10 March	17 April
Malaysia	32,365,999	99	28,208	70	7009	217	114	4	18 March	18 March
Myanmar	54,409,800	83	4996	60	199	4	6	0.1	24 March	12 April
Philippines	109,581,078	368	8321	58	13,221	121	842	8	16 March	22 March
Singapore	5,850,342	8358	98,827	≥80	29,364	5024	22	4	26 March	24 March
Thailand	69,799,978	137	18,482	75	3034	43	56	0.8	22 March	25 March
Vietnam	97,338,579	314	7771	73	324	3	0	–	19 March	22 March

**Data sources accessed 21 May 2020**: *Population*, https://www.worldometers.info/world‐population/population‐by‐country/. *GDP*, https://data.worldbank.org/indicator/NY.GDP.PCAP.PP.CD. *COVID‐19 cases*, https://www.worldometers.info/coronavirus/#countries. *Lockdown*, https://www.kaggle.com/jcyzag/covid19‐lockdown‐dates‐by‐country; https://www.straitstimes.com/asia/se‐asia/coronavirus‐brunei‐reports‐5‐more‐cases‐bringing‐total‐to‐six; https://laotiantimes.com/2020/03/10/weddings‐social‐and‐cultural‐events‐canceled‐amid‐covid‐19‐concerns/; https://en.wikipedia.org/wiki/COVID‐19_pandemic_in_Myanmar. *Border closures*, https://www.aljazeera.com/news/2020/03/coronavirus‐travel‐restrictions‐border‐shutdowns‐country‐200318091505922.html; https://www.businesstraveller.com/business‐travel/2020/03/26/thailand‐closes‐border‐bans‐entry‐for‐foreigners/; https://www.vietnam‐briefing.com/news/covid‐19‐vietnam‐travel‐updates‐restrictions.html/; https://www.straitstimes.com/opinion/an‐unprecedented‐border‐closure‐in‐unprecedented‐times; https://en.wikipedia.org/wiki/COVID‐19_pandemic_in_the_Philippines#cite_note‐291; https://restrictions.info/tr/list/l/; https://en.wikipedia.org/wiki/Travel_restrictions_related_to_the_COVID‐19_pandemic#cite_note‐28. GDP, gross domestic product by purchasing power parity in current international Dollars; UHC, universal health coverage.

^a^ The WHO UHC service coverage index uses a scale of 0 to 100, and was constructed from sub‐indices representing four categories of reproductive, maternal and child health, infectious diseases, non‐communicable diseases, and service capacity and access. Indices described here are from the 2015 baseline analysis, and may not reflect health service coverage for migrant and refugee populations.

Inter‐ and intra‐regional differences in infection patterns have been similarly observed in other epidemics. Following the outbreak of SARS, caused by SARS‐CoV‐1, in China in 2003, there were nearly 500 cases in Canada (mostly in a single province) and 238 cases in Singapore, but only five in neighbouring Malaysia [2]. During the 2009 influenza A (H1N1) pandemic, there were an estimated 5.6 million cases in Italy and 60 million cases in the US [3], but less than 12,000 in Vietnam [4]. To interpret these patterns and define appropriate responses, we can reflect back on key lessons we learned in effectively responding to HIV: *know your epidemic* and *know your response*.

Knowing your epidemic involves working to improve understanding of local epidemic dynamics, including the distribution of risks and parameterizing mathematical models. Recent advancements in data science have allowed unprecedented real‐time access to data that we are using to monitor national COVID‐19 trajectories [5], disaggregate risks for infection and death [6], and track the stringency of government response efforts [7]. However, these data can be considered in the context of historical variability in the trajectories of past respiratory pathogens that may help explain current heterogeneity observed in COVID‐19 case burden. For example global influenza surveillance was scaled up after the 2009 pandemic through the World Health Organization’s FluNet program, and has facilitated extensive research around transmission dynamics as well as associated morbidity and mortality. The current burden of influenza infections tends to be focused within seasonal outbreaks in temperate climates, but may have single or dual peaks with background activity in tropical areas [8]. Even within individual countries, the intensity and frequency of influenza transmission varies by latitude and population characteristics [9]. It is this heterogeneity that epidemiologists and policy makers have come to appreciate when informing the implementation of influenza vaccination campaigns and appear to similarly affect differential COVID‐19 pandemic patterns by region and sub‐region [10].

Knowing your epidemic further suggests the need to understand temporal changes to COVID‐19 and differences within and across countries in order to develop effective control measures. Across the network of 10 countries under the Association of South East Asian Nations (ASEAN), as of 21 May, there had been 72,622 reported cases and 2283 deaths (Table 1) among 667 million people [5]. While the estimated burden of cases and mortality are subject to change and to under‐ascertainment due to limited testing and attribution of mortality, hospitals including intensive care infrastructure have so far generally been able to address COVID‐19 clinical needs.

Multiple hypotheses have been presented to explain these differences compared to the staggering burden of disease in certain epicentres across Western Europe and North America, including social factors such as wearing masks, care practices for the elderly, population age distributions, environment, and pre‐existing immunity to coronaviruses [10]. Importantly, the relatively smaller overall COVID‐19 epidemics in Southeast Asia have not precluded micro‐epidemics, including among those in congregate living settings such as migrant work camps, refugee camps, long‐term care facilities, homeless shelters, and prisons. This concentration of risks is similarly consistent with HIV, where intersecting individual, network, and structural risks impact both the acquisition and transmission of HIV.

The timing and scope of COVID‐19 public health responses have played key roles in regional pandemic control. Consistent with knowing your response, community and government‐led interventions have varied in intensity and breadth across Southeast Asia [11]. However, governments have largely been proactive in their social and physical distancing requirements, which have usually included requiring people to wear masks in public and restricting travel and tourism (Table 1). Knowing your response further means moving away from a uniform approach to managing COVID‐19. Specifically, the ability to empathize and therefore understand that different people need different responses at different times and the dynamics of their risks is essential to an evidence‐based and rights‐affirming response.

For COVID‐19, this also means appreciating that resources to support social distancing requirements should be distributed equitably to those who need them most – such as those living in extreme poverty and migrant workers, refugees, and prisoners. In our primarily low‐ and middle‐income country contexts, “working from home” is a luxury that only a minority of people can afford. As Southeast Asian countries emerge from lockdown and travel restrictions, and COVID‐19 cases potentially resurge, addressing the insufficiencies of our social safety nets is central to implementing pragmatic responses. We also need to sustain the viability of public health and clinical systems to manage competing health priorities, including vaccination, reproductive health, HIV, tuberculosis, acute and chronic non‐communicable conditions, and mental health.

The current and expected future waves of COVID‐19 represent a rapidly emerging threat to the world’s public health, which will likely continue to manifest with substantial heterogeneity within and across countries and populations. Governments in Southeast Asia have imposed broad and sometimes punitive lockdowns, in part because of the limited data available to develop a more refined strategy [12, 13]. In order to strike an optimal balance between COVID‐19 prevention and mitigation, we encourage leveraging a well‐established framework of knowing your epidemic and knowing your response to facilitate rapid transition towards community and government‐led intervention strategies that are impactful, equitable, and contextually appropriate.

## COMPETING INTERESTS

AHS has received travel and grant funding to her institution from ViiV Healthcare.

## AUTHORS’ CONTRIBUTIONS

AHS, NP, SB and AK developed the idea for the Viewpoint, and then wrote and revised it together. All authors have read and approved the final manuscript.
